# Elevated mean platelet volume in oral lichen planus and increased blood urea nitrogen level in its red-form: an observational study

**DOI:** 10.1186/s12903-021-01659-0

**Published:** 2021-06-16

**Authors:** Hui Yao, Yiwen Deng, Guanhuan Du, Yufeng Wang, Guoyao Tang

**Affiliations:** 1 Department of Oral Medicine, Shanghai Ninth People’s Hospital, Shanghai Jiao Tong University School of Medicine, 639 Zhizaoju Road, Shanghai, 200011 China; 2 College of Stomatology, Shanghai Jiao Tong University, Shanghai, China; 3 National Center for Stomatology, Shanghai, China; 4 National Clinical Research Center for Oral Diseases, Shanghai, China; 5 Shanghai Key Laboratory of Stomatology, Shanghai, China

**Keywords:** Lichen planus, Oral blood platelets, Mean platelet volume (MPV), Blood urea nitrogen (BUN), Kidney diseases

## Abstract

**Background:**

This retrospective observational study aims to assess platelet count, mean platelet volume (MPV), blood biochemical tests for liver and kidney function in Chinese oral lichen planus (OLP) patients.

**Methods:**

Eighty pathologically confirmed OLP patients and 51 healthy controls were enrolled. Data on full blood count and biochemical tests were obtained from the electronic medical record system of the hospital.

**Results:**

MPV was elevated in OLP patients compared to controls (10.68 ± 0.97 fL versus 10.33 ± 0.89 fL, *P* = 0.042) while platelet count showed no difference between them. Red-form OLP group had increased blood urea nitrogen (BUN, 5.24 ± 1.15 mmol/L versus 4.69 ± 0.98 mmol/L, *P* = 0.036) than white-form OLP group. By contrast, there were no differences between those two groups in the other variables including MPV, alanine aminotransferase (ALT), aspartate aminotransferase (AST), and creatinine. In terms of C-reactive protein (CRP), 92.5% of the OLP patients had a value of less than 3.48 mg/L. Besides, 75% of the OLP patients were overweight with body mass index (BMI) more than 25 kg/m^2^.

**Conclusions:**

These findings indicate MPV might play roles in inflammation in OLP. The red-form OLP might be associated with damage or reduction of kidney function.

## Background

Oral lichen planus (OLP) is a chronic T-lymphocyte mediated inflammatory disorder, which presents in two primary clinical categories: white forms (reticular, papular, plaquelike) and red forms (erosive, atrophic, bullous) [1]. It is one of the most common diseases in oral medicine clinics. The prevalence of OLP in the normal adult population is 0.49–1.43% [2]. The most serious complication of OLP is oral squamous cell carcinoma. González-Moles et al. reported its malignant transformation rate reached 1.14% [3].

Platelets can interact with leukocytes, secreting inflammatory mediators such as cytokines and chemokines to modulate inflammatory processes in injury or infection state besides its primary hemostasis function [4–6]. Mean platelet volume (MPV) represents the average size of platelet, reflects the platelet stimulation and production rate, and indicates the platelet function and activation for inflammation [7]. Long-lasting inflammation leads to liver damage [5] and kidney disease [8]. Chronic inflammation is the cause of secondary injury to organs gradually, including liver and kidney, possibly via inducing oxidative stress [9].

Some studies showed platelet count and/or MPV alterations in some chronic inflammatory disorders, such as psoriasis [10] and systemic lupus erythematosus (SLE) [11]. Besides, a possible association between those disorders and abnormalities of the liver [12, 13] and kidney function was described previously [14–16]. Some research suggested an association between OLP and hepatitis C virus infection [17, 18]. It is suggested that cytotoxic immune response to the oral epithelial cells infected with HCV makes OLP pathological process happen.

OLP is one type of chronic inflammatory disorders. Platelets might be a potential therapeutic target for OLP, and it might be a risk factor for liver and kidney diseases. However, OLP patients’ platelet parameters and biochemical features of liver and kidney function have not been well studied. We hypothesize some alteration of platelet parameters, liver and kidney function in OLP. Therefore, in this study, we aim to analyze the levels of platelet count, MPV, and liver and kidney function-related parameters such as alanine aminotransferase (ALT), aspartate aminotransferase (AST), blood urea nitrogen (BUN), and creatinine in Chinese patients with OLP.

## Methods

### Population and study samples

This retrospective observational study was conducted in the Department of Oral Medicine, Shanghai Ninth People’s Hospital, Shanghai Jiao Tong University School of Medicine. The process of OLP patient recruitment was based on the electronic medical record system of our hospital between January 2016 and August 2017. Our inclusion criteria were patients aged over 18 years old and clinically diagnosed with OLP, who had not been on any immunomodulatory medication such as steroids in the last 3 months. Exclusion criteria included any underlying systemic disease such as atherosclerotic disease, blood disorder, cancer, infection, inflammation, metabolic syndrome [19]. Exclusion criteria contained GvHD-related, medication-related and dental-material-related oral lichenoid lesions as well. We additionally excluded the patients without biopsy or agreement between clinical and pathological diagnosis. Also, gender- and age-matched healthy subjects served as controls, who were the patients in our clinic with oral fibroma but without any systemic disease. The study protocol was approved by the Ethics Committee of our hospital (No. 2016–1).

### Definition and measurements

Clinical OLP and final pathologically proved OLP were defined according to Modified WHO diagnostic criteria of OLP [20]. We collected participants' demographic data, clinical, and laboratory characteristics available in the database of our hospital. The information included gender, age, clinical type, disease duration, body mass index (BMI), C-reactive protein (CRP), white blood cell (WBC) count, neutrophil count, lymphocyte count, platelet count, MPV, ALT, AST, BUN, and creatinine at their first or second visit before our medication use. We categorized reticular, papular, plaquelike type into white forms and categorized erosive, atrophic, bullous type into red forms. Besides, neutrophils/lymphocytes ratio and platelets/lymphocytes ratio were calculated. CRP data were obtained via a conventional CRP test. It was unable to measure down to the value of less than 3.48 mg/L in our laboratory. We used the reference ranges from our laboratory as the normal values, although there could be some differences in the results between laboratories. Once multiple tests were found, we selected the very blood test when we collected the clinical details.

### Statistical analysis

Each continuous variable was expressed as mean ± standard deviation (SD) or median (interquartile range, IQR). Every categorical document was expressed as number and percentages (%). A chi-squared test was applied to compare Gender variable and a Mann–Whitney test was applied for non-normally distributed continuous data. A *P *value less than 0.05 was considered statistically significant. Each statistical analysis was performed using GraphPad Prism 8 (GraphPad Software, Inc.).

## Results

### Clinical characteristics of subjects

**Fig. 1:**
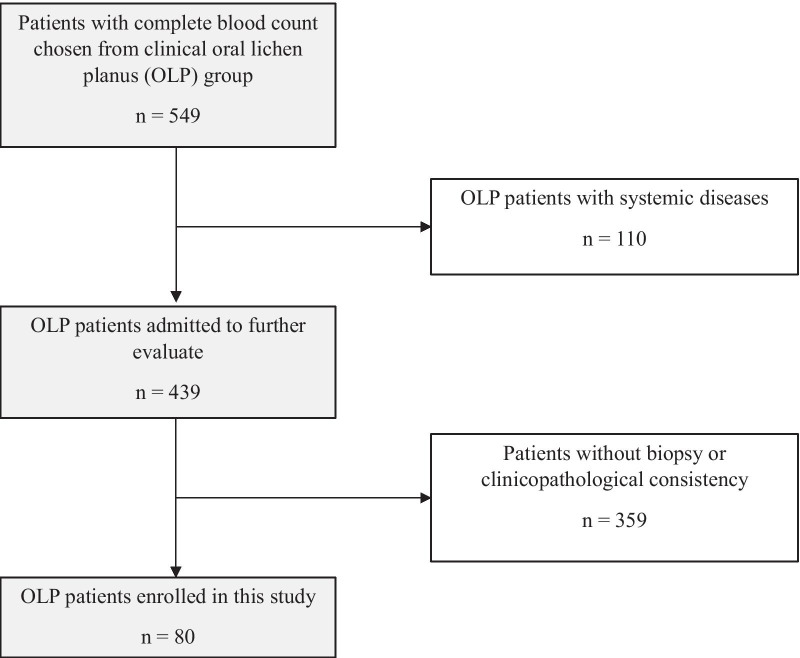
Flow chart

As shown in Fig. 1, there were 549 adult patients diagnosed with clinical OLP. However, 110 out of the 549 OLP patients had some systemic diseases. Table 1 shows the spectrum of the systemic diseases of the 110 OLP patients. The related diseases encompassed atherosclerotic disease (*n* = 2, 1.82%), blood disorder (*n* = 56, 50.91%), cancer (*n* = 3, 2.73%), infection (n = 6, 5.45%), inflammation (*n* = 8, 7.27%) and metabolic syndrome (*n* = 35, 31.82%). Since all those systemic diseases could alter blood counts and/or liver and kidney function, we excluded them from this study. Out of the 549 patients, 359 without biopsy or with other pathological diagnoses were also excluded because their clinical diagnoses might be or were inconsistent with their pathological diagnoses. In the end, a total of 80 pathologically confirmed OLP patients without any systemic disease were analyzed.

**Table 1:** Spectrum of systemic diseases in patients with clinical oral lichen planus

Subjects	*n*	%
**Atherosclerotic disease**	**2**	**1.82**
Coronary heart disease	1	0.91
Rheumatic heart disease on warfarin	1	0.91
**Blood disorders**	**56**	**50.91**
Neutropenia	9	8.18
Neutropenia and thrombrocytopenia	2	1.82
Neutrophilia	13	11.82
Thrombrocytopenia	24	21.82
Thrombrocytosis	8	7.27
**Cancer**	**3**	**2.73**
Breast cancer	1	0.91
Kidney cancer	1	0.91
Thymoma	1	0.91
**Infection**	**6**	**5.45**
HBV	3	2.73
HCV	2	1.82
Tuberculosis	1	0.91
**Inflammation**	**8**	**7.27**
Arthritis	1	0.91
Bronchitis	1	0.91
Emphysema	1	0.91
Hashimoto thyroiditis	3	2.73
Hashimoto thyroiditis and dyslipidemia	1	0.91
Hashimoto thyroiditis and stroke	1	0.91
**Metabolic syndrome**	**35**	**31.82**
Diabetes mellitus	5	4.55
Dyslipidemia	1	0.91
Gout	1	0.91
Hypertension	18	16.36
Hypertension and diabetes mellitus	8	7.27
Hypertension and dyslipidemia	1	0.91
Hypertension and stroke	1	0.91

The bold value was composed of atherosclerotic disease, blood disorder, cancer, infection, inflammation, metabolic syndrome. These systemic diseases were excluded in this study. Here, the information showed the systemic background in the patients with oral lichen planus

Clinical details are shown in Table 2 for both OLP patient group (*n* = 80) and healthy control group (*n* = 51). We attempted to match the gender ratio (44 women and 36 men versus 32 women and 19 men, OLP versus control, respectively) and the age ranges (median 43 (IQR, 35.25–55) years versus median 42 (IQR, 33–51) years, OLP versus control, respectively) to minimize their impact on blood counts and blood biochemistries. Due to limited clinical information recorded, 72 out of the 80 OLP patients with clinical type information were further divided into white-form OLP (WF-OLP, *n* = 31) and red-form OLP (RF-OLP, *n* = 41). Meanwhile, among the 80 patients, the median duration of 66 OLP patients with recorded details was 4.5 (IQR, 2–6.25) months.

**Table 2:** Clinical details of oral lichen planus patients and healthy controls

	OLP		Control		*P *value
	*n*	Result	*n*	Result	
Total number	80		51		
Gender, n (%)					0.381
Male	36	45	19	37.25	
Female	44	55	32	62.75	
Age, median (IQR) years	80	43 (35.25 – 55)	51	42 (33 – 51)	0.18
Clinical type, n (%)					
Red-form	41	51.25			
White-form	31	38.75			
Not recorded	8	10			
Duration, median (IQR) months	66	4.5 (2 – 6.25)			
CRP, n (%)					
≥ 3.48 mg/L	6	7.5			
< 3.48 mg/L	74	92.5			
BMI, mean (± SD) kg/m^2^	64	23.45 ± 3.29			

A chi-squared test was applied for Gender variable; a Mann–Whitney test was employed for Age variable between two groups

BMI, body mass index; CRP, C-reactive protein; IQR, interquartile range; OLP, oral lichen planus; SD, standard deviation

The 80 OLP patients were divided into two categories—CRP ≥ 3.48 mg/L and CRP < 3.48 mg/L. The majority of the OLP patients (*n* = 74, 92.5%) had a CRP value of less than 3.48 mg/L. Since body mass index (BMI) could be one factor which associates with inflammatory diseases, we analyzed BMI from 64 OLP patients with the related information recorded. Their mean value was 23.45 ± 3.29 kg/m^2^ (*n* = 64). Out of the 64 patients, 48 (75%) were categorized into overweight group (BMI ≥ 25 kg/m^2^); 15 (23.44%) into normal weight (BMI < 25 kg/m^2^ but > 18.5 kg/m^2^); 1 (1.56%) into underweight (BMI ≤ 18.5 kg/m^2^) (Data no show).

### Platelet parameters in OLP

Full blood count records were analyzed from OLP (*n* = 80) and control subjects (*n* = 51) to access the platelet parameters. As shown in Table 3, MPV of patients with OLP was significantly higher than that of healthy controls (10.68 ± 0.97 fL versus 10.33 ± 0.89 fL; *P* = 0.042). Since we divided the patients into RF-OLP group and WF-OLP group, MPV from those two groups was analyzed as well. As shown in Table 4, we had not found MPV differences between those two types of OLP (10.44 ± 0.91 fL versus 10.85 ± 1.00 fL, *P* = 0.072). Also, there was no significant difference in platelet count between those two groups ((221.3 ± 48.94) × 10^9^/L versus (228.8 ± 44.47) × 10^9^/L, *P* = 0.376). We detected no significant differences (*P* > 0.05) in white blood cells, neutrophils, lymphocytes, neutrophils/lymphocytes ratio, and platelets/lymphocytes ratio between OLP patient group and healthy control group (Table 3).

**Table 3:** Hematological tests of oral lichen planus patients and healthy controls

	OLP		Control		Reference ranges	*P *value
	*n*	Result	*n*	Result		
Total number	80		51			
WBC, 10^9^/L	80	5.84 ± 1.28	51	6.06 ± 1.45	3.5 – 9.5	0.349
Neutrophils, 10^9^/L	80	3.38 ± 1.11	51	3.63 ± 1.22	1.8 – 6.3	0.230
Lymphocytes, 10^9^/L	80	1.91 ± 0.58	51	1.84 ± 0.50	1.1 – 3.2	0.505
Platelets, 10^9^/L	80	221.3 ± 48.94	51	228.8 ± 44.47	125 – 350	0.376
MPV, fl	79	10.68 ± 0.97	51	10.33 ± 0.89	8 – 12.5	*0.042*
Neu/Lym ratio	80	1.98 ± 1.13	51	2.11 ± 1.07		0.507
Pla/Lym ratio	80	125.6 ± 45.30	51	130.4 ± 34.17		0.513

Data are presented as number or mean (± SD)

Lym, lymphocytes; MPV, mean platelet volume; Neu, neutrophils; OLP, oral lichen planus; Pla, platelets; WBC, white blood cell

A *P *value is estimated by the Mann–Whitney test

**Table 4:** Mean platelet volume, liver and kidney tests in oral lichen planus patients

	White-form OLP		Red-form OLP		Reference ranges	*P *value
	*n*	Result	*n*	Result		
Total number	31		41			
MPV, fL	31	10.44 ± 0.91	40	10.85 ± 1.00	8 – 12.5	0.072
ALT, U/L	24	25.54 ± 20.57	33	20.58 ± 10.72	10 – 49	0.241
AST, U/L	24	23.54 ± 11.65	33	20.97 ± 5.56	0 – 34	0.272
BUN, mmol/L	29	4.69 ± 0.98	40	5.24 ± 1.15	2.5 – 6.4	*0.036*
Creatinine, μmol/L	30	88.00 ± 11.53	41	88.59 ± 15.36	62 – 115	0.855

Data are presented as number or mean (± SD)

BUN, blood urea nitrogen; ALT, alanine aminotransferase; AST, aspartate aminotransferase; MPV, mean platelet volume; OLP, oral lichen planus

A *P *value is estimated by the Mann–Whitney test

### Liver and kidney function in OLP

Owning to a lack of controls’ liver and kidney tests, we designed to compare their difference between RF-OLP group and WF-OLP group. As shown in Table 4, BUN was significantly increased in RF-OLP patients compared with that in WF-OLP patients ((5.24 ± 1.15) mmol/L versus (4.69 ± 0.98) mmol/L, *P* = 0.036). Despite that difference, there were no statistical differences (*P* > 0.05) between them in other variables such as ALT, AST, and creatinine (Table 4), and all parameters tested were within the normal ranges of healthy individuals based on clinical test references.

## Discussion

In this study, we found that elevated MPV in Chinese patients with OLP despite no significant difference in platelet count. Although very few studies of OLP on MPV are present, a previous study conducted by Ozlu et al. found that the dermal lichen planus group had significantly lower MPV compared to the healthy control group [21], which was different with our finding. This discrepancy might be due to the material and methods used in the studies, such as different sites of the lesion, whether the patients included were biopsy-confirmed or any other factors influencing the results. On the contrary, our finding is identical to a series of other chronic inflammatory conditions including psoriasis and SLE. Canpolat and his colleagues demonstrated that MPV significantly increased in patients with psoriasis than in control subjects [10]. Yavuz’s team found that MPV was statistically elevated in juvenile SLE patients than in controls [22]. It was also confirmed in the adult patients with SLE [11].

MPV reflects platelet's activation. Platelets play an important role in the acquired immune responses via CD40L signaling [23, 24]. Furthermore, some scientists noted intraepithelial T cells in OLP lesions expressing CD40 and CD40L rather than basal keratinocyte through immunohistochemistry and in situ mRNA hybridization [25]. The following research also showed the expression of CD40 was increased by IFN-gamma stimulation in OLP [26]. Therefore, the underlying mechanism might be that MPV reacts and increases after platelet apoptosis, platelet particle release [27], by which the immune system needs platelet activation and their involvement in the inflammatory process. Although other studies had divided views on altered patterns of MPV, the possible reason is complex. To provide more evident support, further research needs to be conducted.

We have also observed a higher level of BUN in RF-OLP group compared to WF-OLP group. Elevated urea level is common in renal impairment and tends to be a signal parameter in kidney injury, including chronic kidney disease (CKD). Our finding in this exploratory study is similar to the results in psoriasis and SLE studies. It is understood that around 50% of SLE patients are associated with renal involvement, which is known as lupus nephritis [28]. A French research team concluded that chronic kidney disease (CDK) contributed greatly to overall morbidity and mortality in SLE [29]. In terms of psoriasis, Wan and his colleagues found that there was a high risk of CDK in patients with moderate to severe psoriasis [16]. In 2015, Chi et al. confirmed that advanced psoriasis was a risk factor of incident CKD [30].

The potential reason for kidney injury might be due to autoimmunity, drug-related toxicity, or chronic inflammation [15]. In this study, we tried to minimize the effects of medications by adopting strict inclusion criteria where immunomodulatory drugs were not in use for three months. Furthermore, there is no identified specific antigen or lichen planus antigen involved in its pathological mechanism of OLP so that it is inappropriate to categorize it as a genuine autoimmune disease [31]. On the contrary, it is safe to say OLP undergoes a cell-mediated immune response. Therefore, the most possible explanation for the BUN rise is chronic inflammation. Chronic inflammation and proinflammatory cytokines, such as TNF-alpha are the possible links between active inflammation and kidney damage [32–34]. The underlying mechanism might be related to vitamin D deficiency [35, 36], which plays a crucial role in multiple functions such as regulating blood pressure, modulating immune process, and influencing cell growth [15]. However, new and further studies should be designed and help elucidate the possible mechanism [30].

Although this single-center retrospective observational study included a relatively large sample size of the OLP patients with biopsy-confirmed, it still has some limitations due to its retrospective nature. Initially, it was not created for research. A few parameters are not sensitive, precise enough, or even vacant, especially CRP data. This study also needs longitudinal observation data on the influence of treatment on OLP patients. Further research needs to enroll a larger population of OLP patients from multiple oral medicine clinics and to be designed more strictly to decrease the influence of the limitations mentioned above. The exact mechanism to explain the association of MPV and BUN with OLP should be conducted as well.

## Conclusions

This study has demonstrated that MPV is elevated in OLP patients, and RF-OLP group has higher BUN than WF-OLP patients. These findings suggest that MPV might play vital roles in the inflammatory mechanism in OLP and RF-OLP might be associated with the functional damage or reduction of the kidney.

## Data Availability

The data and materials collected in this research are available from the corresponding author when requested reasonably.

## Abbreviations

GvHD: Graft-versus-host disease

## References

[1] Le Cleach L, Chosidow O (2012). Clinical practice. Lichen planus. N Engl J Med.

[2] González-Moles MÁ, Warnakulasuriya S, González-Ruiz I, González-Ruiz L, Ayén Á, Lenouvel D, Ruiz-Ávila I, Ramos-García P (2020). Worldwide prevalence of oral lichen planus: a systematic review and meta-analysis. Oral Dis.

[3] González-Moles MÁ, Ruiz-Ávila I, González-Ruiz L, Ayén Á, Gil-Montoya JA, Ramos-García P (2019). Malignant transformation risk of oral lichen planus: a systematic review and comprehensive meta-analysis. Oral Oncol.

[4] Diacovo TG, Puri KD, Warnock RA, Springer TA, von Andrian UH (1996). Platelet-mediated lymphocyte delivery to high endothelial venules. Science.

[5] Iannacone M, Sitia G, Isogawa M, Marchese P, Castro MG, Lowenstein PR, Chisari FV, Ruggeri ZM, Guidotti LG (2005). Platelets mediate cytotoxic T lymphocyte-induced liver damage. Nat Med.

[6] Morrell CN, Aggrey AA, Chapman LM, Modjeski KL (2014). Emerging roles for platelets as immune and inflammatory cells. Blood.

[7] Gasparyan AY, Ayvazyan L, Mikhailidis DP, Kitas GD (2011). Mean platelet volume: a link between thrombosis and inflammation?. Curr Pharm Des.

[8] Linge P, Fortin PR, Lood C, Bengtsson AA, Boilard E (2018). The non-haemostatic role of platelets in systemic lupus erythematosus. Nat Rev Rheumatol.

[9] Furman D, Campisi J, Verdin E, Carrera-Bastos P, Targ S, Franceschi C, Ferrucci L, Gilroy DW, Fasano A, Miller GW (2019). Chronic inflammation in the etiology of disease across the life span. Nat Med.

[10] Canpolat F, Akpinar H, Eskioğlu F (2010). Mean platelet volume in psoriasis and psoriatic arthritis. Clin Rheumatol.

[11] Bai M, Xing L, Feng J, Cui C, Huang L, Liang G (2016). Mean platelet volume could reflect disease activity of adult patients with systemic lupus erythematosus. Clin Lab.

[12] Finet A, Viguier M, Chazouillères O, Amatore F, Paul C, Richard MA, Chosidow O, Bachelez H, Sbidian E (2016). Liver test abnormalities in patients admitted for severe psoriasis: prevalence and associated risk factors. J Eur Acad Dermatol Venereol JEADV.

[13] Liu Y, Yu J, Oaks Z, Marchena-Mendez I, Francis L, Bonilla E, Aleksiejuk P, Patel J, Banki K, Landas SK (2015). Liver injury correlates with biomarkers of autoimmunity and disease activity and represents an organ system involvement in patients with systemic lupus erythematosus. Clin Immunol.

[14] Koubar SH, Kort J, Kawtharani S, Chaaya M, Makki M, Uthman I (2019). Characteristics of lupus and lupus nephritis at a tertiary care center in Lebanon. Lupus.

[15] Visconti L, Leonardi G, Buemi M, Santoro D, Cernaro V, Ricciardi CA, Lacquaniti A, Coppolino G (2016). Kidney disease and psoriasis: novel evidences beyond old concepts. Clin Rheumatol.

[16] Wan J, Wang S, Haynes K, Denburg MR, Shin DB, Gelfand JM (2013). Risk of moderate to advanced kidney disease in patients with psoriasis: population based cohort study. BMJ.

[17] Petti S, Rabiei M, De Luca M, Scully C (2011). The magnitude of the association between hepatitis C virus infection and oral lichen planus: meta-analysis and case control study. Odontology.

[18] Alaizari NA, Al-Maweri SA, Al-Shamiri HM, Tarakji B, Shugaa-Addin B (2016). Hepatitis C virus infections in oral lichen planus: a systematic review and meta-analysis. Aust Dent J.

[19] Qin B, Ma N, Tang Q, Wei T, Yang M, Fu H, Hu Z, Liang Y, Yang Z, Zhong R (2016). Neutrophil to lymphocyte ratio (NLR) and platelet to lymphocyte ratio (PLR) were useful markers in assessment of inflammatory response and disease activity in SLE patients. Mod Rheumatol.

[20] van der Meij EH, van der Waal I (2003). Lack of clinicopathologic correlation in the diagnosis of oral lichen planus based on the presently available diagnostic criteria and suggestions for modifications. J Oral Pathol Med.

[21] Ozlu E, Karadag AS, Toprak AE, Uzuncakmak TK, Gerin F, Aksu F, Ozakpınar O, Akdeniz N (2016). Evaluation of cardiovascular risk factors, haematological and biochemical parameters, and serum endocan levels in patients with lichen planus. Dermatology (Basel).

[22] Yavuz S, Ece A (2014). Mean platelet volume as an indicator of disease activity in juvenile SLE. Clin Rheumatol.

[23] Danese S, Katz JA, Saibeni S, Papa A, Gasbarrini A, Vecchi M, Fiocchi C (2003). Activated platelets are the source of elevated levels of soluble CD40 ligand in the circulation of inflammatory bowel disease patients. Gut.

[24] Elgueta R, Benson MJ, de Vries VC, Wasiuk A, Guo Y, Noelle RJ (2009). Molecular mechanism and function of CD40/CD40L engagement in the immune system. Immunol Rev.

[25] Neppelberg E, Loro LL, Oijordsbakken G, Johannessen AC (2007). Altered CD40 and E-cadherin expression–putative role in oral lichen planus. J Oral Pathol Med.

[26] Marshall A, Celentano A, Cirillo N, Mirams M, McCullough M, Porter S (2017). Immune receptors CD40 and CD86 in oral keratinocytes and implications for oral lichen planus. J Oral Sci.

[27] Boilard E, Nigrovic PA, Larabee K, Watts GFM, Coblyn JS, Weinblatt ME, Massarotti EM, Remold-O'Donnell E, Farndale RW, Ware J (2010). Platelets amplify inflammation in arthritis via collagen-dependent microparticle production. Science.

[28] Almaani S, Meara A, Rovin BH (2017). Update on lupus nephritis. Clin J Am Soc Nephrol.

[29] Mageau A, Timsit J-F, Perrozziello A, Ruckly S, Dupuis C, Bouadma L, Papo T, Sacre K (2019). The burden of chronic kidney disease in systemic lupus erythematosus: a nationwide epidemiologic study. Autoimmun Rev.

[30] Chi C-C, Wang J, Chen Y-F, Wang S-H, Chen F-L, Tung T-H (2015). Risk of incident chronic kidney disease and end-stage renal disease in patients with psoriasis: a nationwide population-based cohort study. J Dermatol Sci.

[31] McCartan BE, Lamey P (2000). Lichen planus–specific antigen in oral lichen planus and oral lichenoid drug eruptions. Oral Surg Oral Med Oral Pathol Oral Radiol Endod.

[32] Davidovici BB, Sattar N, Prinz JC, Jörg PC, Puig L, Emery P, Barker JN, van de Kerkhof P, Ståhle M, Nestle FO (2010). Psoriasis and systemic inflammatory diseases: potential mechanistic links between skin disease and co-morbid conditions. J Investig Dermatol.

[33] Griffiths CE, Barker JN (2007). Pathogenesis and clinical features of psoriasis. Lancet.

[34] Nestle FO, Kaplan DH, Barker J (2009). Psoriasis. N Engl J Med.

[35] Gisondi P, Rossini M, Di Cesare A, Idolazzi L, Farina S, Beltrami G, Peris K, Girolomoni G (2012). Vitamin D status in patients with chronic plaque psoriasis. Br J Dermatol.

[36] Liu D, Fang Y-X, Wu X, Tan W, Zhou W, Zhang Y, Liu Y-Q, Li G-Q (2019). 1,25-(OH)D/Vitamin D receptor alleviates systemic lupus erythematosus by downregulating Skp2 and upregulating p27. Cell Commun Signal.

